# Contemporary indications for first-time revision surgery after primary cementless total hip arthroplasty with emphasis on early failures

**DOI:** 10.1186/s13018-021-02298-5

**Published:** 2021-02-17

**Authors:** Goro Motomura, Satoshi Hamai, Satoshi Ikemura, Masanori Fujii, Shinya Kawahara, Soichiro Yoshino, Yasuharu Nakashima

**Affiliations:** Department of Orthopaedic Surgery, Graduate School of Medical Sciences, Kyushu University, 3-1-1 Maidashi, Higashi-ku, Fukuoka, 812-8582 Japan

**Keywords:** Cementless total hip arthroplasty, First-time revision surgery, Indication

## Abstract

**Background:**

To clarify contemporary indications for first-time revision surgery after primary cementless total hip arthroplasty (THA) for addressing potential issues with cementless THA.

**Methods:**

Data for 101 consecutive hips in 94 patients who underwent primary cementless THA at our institution and subsequently underwent first-time revision surgery were retrospectively reviewed. Baseline characteristics, indications for first-time revision surgery, and time from the primary THA to first-time revision surgery were evaluated.

**Results:**

The overall mean time to first-time revision surgery was 10.3 years (range, 0–33 years). The indications for first-time revision surgery were polyethylene wear and osteolysis in 33 hips, aseptic loosening in 25 hips, infection in 17 hips, periprosthetic fracture in 13 hips, instability in 8 hips, and component failure (liner dissociation or stem fracture) in 5 hips. Thirty-seven hips (37%) had undergone first-time revision surgery within 5 years of primary THA, of which the most common indications were infection and periprosthetic fracture.

**Conclusion:**

The current results suggested that reducing the number of early failures seems to be essential form improving THA outcomes.

## Background

Total hip arthroplasty (THA) is recognized as one of the most successful surgeries of the twentieth century [1]. The number of surgeries performed each year and degree of durability have been increasing [2–4]. However, there is still potential for postoperative complications such as infection and dislocation despite advances in technology and surgical technique [5]. In addition, osteolysis and aseptic loosening due to bearing surface could occur over time [5]. Given the rise in the popularity of the procedure, the incidence of revision surgery is projected to increase [6].

Early failure requiring revision surgery is an important issue. In 2006, Dobzyniak et al. reported that 39% of patients underwent revision surgery within 5 years of initial THA. Instability was the main indication, followed by aseptic loosening [7]. In 2008, Ulrich et al. showed that the overall mean time to revision surgery at two tertiary referral centers was 83 months and 50% of revision surgeries occurred in less than 5 years. Most procedures were due to instability and deep infection [8]. Recently, Kelmer et al. demonstrated that the overall mean time to revision surgery was 8.51 years and 31% of patients required revision surgery within 2 years of THA, mainly due to mechanical failure and infection, followed by dislocation [9]. These reports remind us of the impact of early failure requiring revision surgery after THA. However, information regarding indications and timing of revision surgery in light of various influencing factors, such as implant-related factors and surgical skills, remains limited [10, 11]. The purpose of this study was to assess indications and timing of first-time revision surgery after primary cementless THA performed at our institution to address potential issues with cementless THA.

## Methods

This study was approved by our institutional review board. The need for informed consent was waived due to the retrospective and anonymous study design. Data for 101 consecutive hips in 94 patients who underwent primary cementless THA at our institution and subsequently underwent first-time revision surgery during 2008–2019 were reviewed. The patients included 27 men and 67 women with a mean age at first-time revision surgery of 68 years (range, 39–88 years) and a mean body mass index (BMI) of 22.9 kg/m^2^ (range, 16.0–36.3 kg/m^2^). Primary diagnosis was osteoarthritis for 72 hips, osteonecrosis for 14 hips, inflammatory arthritis (including rheumatoid arthritis, juvenile idiopathic arthritis, ankylosing spondylitis, and rapidly destructive coxarthrosis) in 8 hips, post-traumatic arthritis in 5 hips, and femoral neck fracture in 2 hips. Previous hip surgery before initial THA had been performed in 23 hips, including femoral osteotomy in 12 hips, pelvic osteotomy or acetabuloplasty in 4 hips, bipolar hemiarthroplasty in 4 hips, fixation of femoral neck fracture in 2 hips, and arthrodesis in 1 hip. Primary cementless THA, including conversion of a bipolar implant to hip arthroplasty, was performed through the posterolateral approach. The capsule, piriformis, and short rotator muscles were repaired as much as possible. Cementless femoral stems were used in all of the initial THA procedures, which consisted of PerFix or PerFix-910 (Kyocera, Kyoto, Japan) in 64 hips and Multilock stem (Zimmer-Biomet, Warsaw, IN, USA) in 13 hips. Various other stems were used in a small number of hips, including TM stem (Zimmer-Biomet) in 4 hips and SROM (Depuy, Warsaw, IN) in 3 hips. Cementless acetabular cups had been used in all of the initial THA procedures, including AMS cup (Kyocera) in 62 hips and HGP ΙΙ cup (Zimmer-Biomet) in 11 hips. Various other cups were also used in a small number of hips, including SQRUM TT cup (Kyocera) in 8 hips and TM cup (Zimmer-Biomet) in 6 hips. Head size varied by time period of surgery and cup size. Head size was 22 mm in 40 hips, 26 mm in 35 hips, and 32 mm in 26 hips. Conventional polyethylene was used until December 1999; conventional polyethylene was used in 52 hips and crosslinked polyethylene was used in 49 hips.

First-time revision surgery was defined as the first therapeutic surgery after THA, including reoperative surgery that did not affect any prosthetic implant, isolated head and liner exchange, and revision with removal or replacement of at least one nonmodular implant (acetabular shell or femoral stem) [12]. The indication for first-time revision surgery was determined based on medical records and preoperative imaging. Indications were classified into one of the following categories: infection, including suspected infection without culture-positive findings; periprosthetic fracture; instability; polyethylene wear and osteolysis; aseptic loosening of the acetabular shell, femoral stem, or both; and component failure. Time from the primary THA to first-time revision surgery and subsequent surgeries after first-time revision surgery were also examined.

Differences in the following characteristics between hips with first-time revision surgery performed within 5 years of THA and first-time revision surgery performed more than 5 years after THA were compared using Fisher’s exact test: gender, age at the time of initial THA (age <50 years or ≥50 years), previous hip surgery, and diagnosis of osteoarthritis. All statistical analyses were performed using the JMP software program (version 15.0, SAS Institute, Cary, NC, USA). *P* values less than 0.05 were considered to be statistically significant.

## Results

**Fig. 1:**
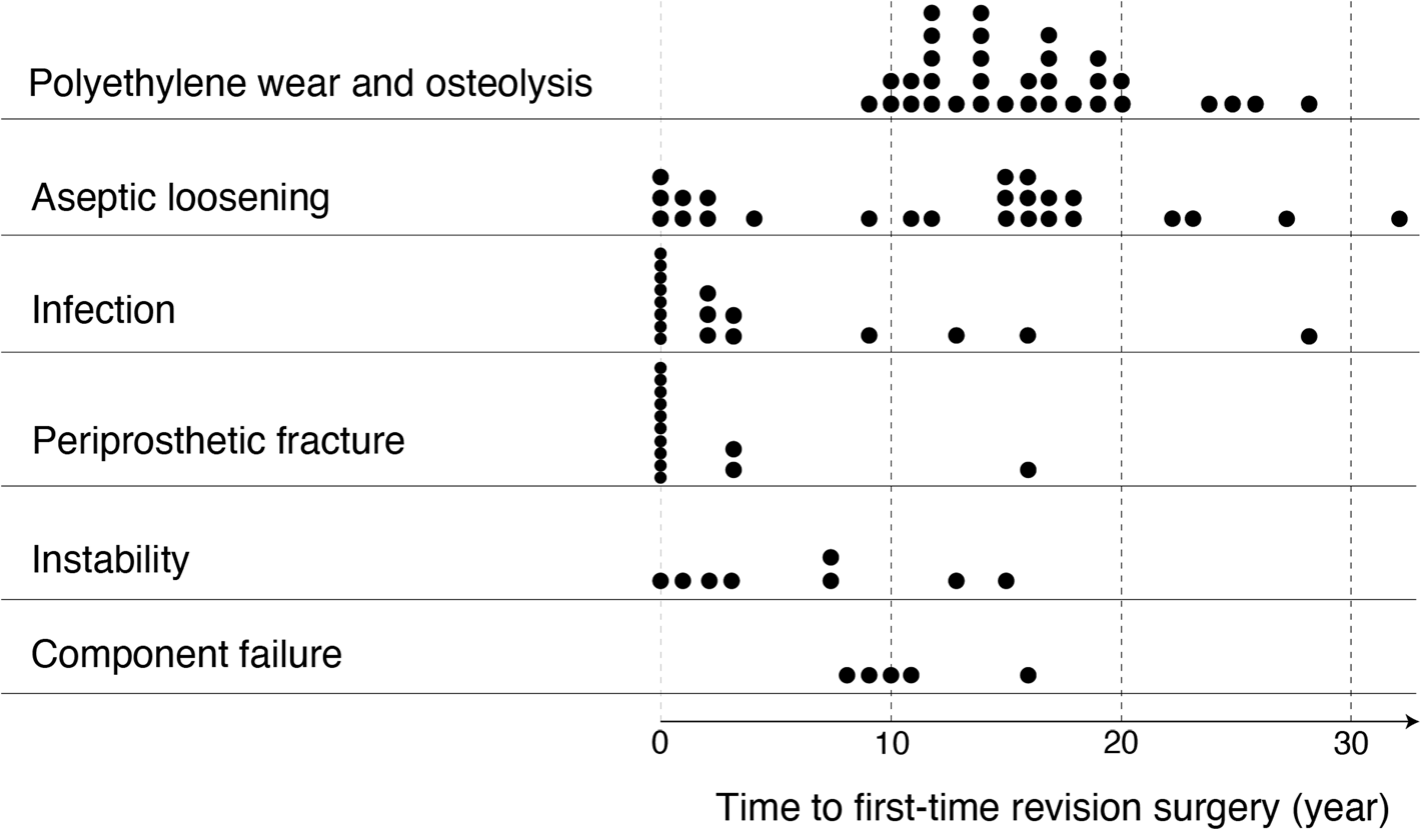
Indications and timing of first-time revision surgery

The overall mean time from primary THA to first-time revision surgery was 10.3 years (range, 0–33 years). The indication for first-time revision surgery was polyethylene wear and osteolysis in 33 hips, aseptic loosening in 25 hips, infection in 17 hips, periprosthetic fracture in 13 hips, instability in 8 hips, and component failure (liner dissociation and femoral stem fracture) in 5 hips (Fig. 1). Thirty-seven surgeries (37%) were performed within 5 years of primary THA, of which the most common indications were infection and periprosthetic fracture. The proportion of males and patients aged over 50 years was significantly higher among hips with first-time revision surgery performed within 5 years of THA than those performed more than 5 years after THA (*p*=0.0027 and *p*=0.0165, respectively) (Table 1).

**Table 1:** Patient characteristics by time from primary THA to first-time revision surgery

	Time from primary THA to first-time revision surgery		*P* value*
	≤5 years (37 hips)	>5 years (64 hips)	
Gender			0.0027
Male	17	11	
Female	20	53	
Age at primary THA, years			0.0165
<50	4	21	
≥50 years	33	43	
Primary diagnosis			0.1706
Osteoarthritis	23	49	
Other	14	15	
Previous surgery			1.0000
Yes	8	15	
No	29	49	

*Fisher’s exact test

First-time revision surgery due to polyethylene wear and osteolysis was performed for 33 hips in 28 patients, at a mean time of 16.0 years (range, 9–28 years) after THA. This group included 3 men and 25 women with a mean age at primary THA of 55 years (range, 28–72 years). All 33 hips that underwent first-time revision surgery consisting of isolated head and liner exchange required no subsequent surgeries. In 32 of 33 hips, conventional polyethylene liner was used for primary THA.

First-time revision surgery due to aseptic acetabular shell loosening was performed for 17 hips in 16 patients at a mean time of 13.6 years (range, 0–27 years) after THA. This group consisted of 4 men and 12 women with a mean age at primary THA of 55 years (range, 29–78 years). First-time revision surgery due to aseptic femoral stem loosening was performed for eight hips in eight patients at a mean time of 9.8 years (range, 0–33 years) after THA. This group consisted of three men and five women with a mean age at primary THA of 54 years (range, 40–73 years). All 25 hips underwent first-time revision surgery consisting of replacement of the acetabular shell (16 hips), femoral stem (6 hips), or both (3 hips). Of these 25 surgeries, 3 were followed by 1 or more subsequent surgeries due to aseptic loosening, infection, or instability. In 15 of 17 hips with first-time revision surgery performed more than 5 years after THA, conventional polyethylene liner was used for primary THA.

First-time revision surgery due to infection was performed for 17 hips in 17 patients at a mean time of 4.6 years (range, 0–28 years) after THA. This group included 10 men and 7 women with a mean age at primary THA of 57 years (range, 33–70 years). Of these 17 hips, 13 (76%) underwent reoperative surgery (6 hips) or isolated head and liner exchange (7 hips) at a mean time of 3.3 years (range, 0–16 years) after primary THA; 5 (38%) of 13 hips required 1 or more subsequent surgeries for recurrence of infection. The remaining four hips (24%) underwent removal surgery at a mean time of 8.8 years (range, 2–28 years) after THA, followed by second-stage replacement surgery.

First-time revision surgeries due to periprosthetic fracture were performed for 13 hips in 13 patients at a mean time of 1.7 years (range, 0–16 years) after THA. This group consisted of 3 men and 10 women with a mean age at primary THA of 71 years (range, 55–85 years). Two femoral fractures classified as Vancouver type A underwent fracture fixation without any implant change. Periprosthetic fracture in 11 hips, including 2 acetabular fractures and 9 femoral fractures classified as Vancouver type B2, underwent replacement of the acetabular shell or femoral stem. None of these hips required subsequent surgery.

First-time revision surgery due to instability was performed for eight hips in eight patients at a mean time of 6.0 years (range, 0–15 years) after THA. This group consisted of two men and six women with a mean age at the primary THA of 66 years (range, 50–84 years). Seven (88%) of eight hips underwent isolated head or liner exchange (six hips) or replacement of acetabular shell (one hip) and required no subsequent surgery. The remaining hip required open reduction due to untreated dislocation. In seven (88%) of eight hips, a femoral head size of 26 mm or smaller was used for primary THA.

First-time revision surgery due to component failure was performed for five hips in five patients at a mean time of 10.8 years (range, 8–16 years) after THA. This group consisted of two men and three women with a mean age at primary THA of 43 years (range, 28–51 years). Two hips in two male patients underwent revision with replacement of the femoral stem for stem fracture. The remaining three hips underwent isolated head and liner exchange (two hips) or replacement of the acetabular shell (one hip) for polyethylene liner breakage. None of the hips required subsequent surgery.

## Discussion

This retrospective study showed the contemporary indications and timing of first-time revision surgery after primary cementless THA in a university hospital. Early revision, within 5 years of THA, accounted for 37% of all revision cases; infection and periprosthetic fracture were common indications. Although THA is certainly recognized as a successful treatment that is expected to improve hip joint function, we have to recognize potential issues with cementless THA again.

While infection is a leading cause of early revision in many cohort studies, periprosthetic fracture is not always the case [7–9]. One reason for the current findings may be that our patients were limited to those who underwent cementless THA, which has been shown to be significantly associated with early periprosthetic fracture [13, 14]. A previous multicenter study from Denmark demonstrated that uncemented femoral components are associated with an increased risk of early periprosthetic femoral fracture, especially in patients who are elderly, female, and have osteoporosis [14]. In the current study, 9 of 10 cases of revision surgery performed within 1 year due to periprosthetic fracture were performed in females with a mean age of 71 years. This finding suggests the need for patient selection and careful surgical technique in order to minimize the number of early fractures.

In a previous nationwide population-based study, male gender was found to be associated with an increased adjusted relative risk of revision [15]. A recent meta-analysis demonstrated that males have an increased risk of revision due to infection after primary THA [16]. In the current series, 10 of 13 patients who underwent early revision due to infection were males, which may have contributed to the proportion of male patients who underwent early revision. Although this study could not establish a link between male gender and infection, we should be aware that male gender may be a possible risk factor for early revision.

A recent study suggested a difference in indications for revision between young and general older population of patients [17]. Kahlenberg et al. demonstrated that, in young patients, acetabular loosening, femur loosening, and polyethylene wear are the most common indications for revision, while instability and infection are less common [17]. Considering that instability and infection are likely to occur soon after initial THA, the low proportion of patients under 50 years of age who underwent early revision in this study may be consistent with their results. In addition, the characteristics of our institution, which often performs joint-preserving surgery for young patients when indicated, may have influenced the current results due to relatively narrow indications for THA in young patients.

Several studies have demonstrated that highly crosslinked polyethylene significantly reduces wear and is associated with lower rate of revision surgery [18–21]. Considering that conventional polyethylene was used in 47 of 50 hips with first-time revision surgery performed more than 5 years after THA due to polyethylene wear and osteolysis or aseptic loosening, the number of procedures could be reduced by the use of crosslinked polyethylene in the future. On the other hand, early failure due to aseptic loosening occurred in eight hips in the current series. A recent study indicated that 50% of early revision cases are potentially avoidable, including cases involving early aseptic loosening [22]. Although it is difficult to find similarities due to the small number of cases, we believe that careful preparation and accurate surgical execution could be essential to reducing the occurrence of early aseptic loosening.

This retrospective study had several limitations. First, the number of cases was small, mainly because this study was limited to patients who had undergone initial cementless THA at our hospital in order to obtain accurate information from the time of initial THA. However, all initial THA procedures in the current series were performed in the same way by experienced surgeons. Given the advantage of reduced bias based on referral patterns, we believe that this study is valuable. Second, we could not assess the incidence rate of revision surgery for initial THA because this was a case series study of revision surgery performed between 2008 and 2019. Third, multiple implants have been used due to the long period of time when initial THA procedures occurred. Therefore, examining whether the cause of revision originates from the implant was not possible in this study. A future prospective study is needed for clarifying possible effects of implants on the need for revision surgery.

## Conclusion

The current results suggested that reducing the number of early failures seems to be an essential form improving THA outcomes.

## Data Availability

The datasets used and/or analyzed during the current study are available from the corresponding author on reasonable request.

## Abbreviations

THA: Total hip arthroplasty
BMI: Body mass index
